# Ion transport through electrolyte/polyelectrolyte multi-layers

**DOI:** 10.1038/srep11583

**Published:** 2015-06-26

**Authors:** Robert Femmer, Ali Mani, Matthias Wessling

**Affiliations:** 1 AVT Chemical Process Engineering, RWTH Aachen University, Turmstr. 46, 52064 Aachen, Germany; 2 Department of Mechanical Engineering, Stanford University, Stanford, CA 94305, USA; 3 DWI, Forckenbeckstr. 50, 52074 Aachen, Germany

## Abstract

Ion transport of multi-ionic solutions through layered electrolyte and polyelectrolyte structures are relevant in a large variety of technical systems such as micro and nanofluidic devices, sensors, batteries and large desalination process systems. We report a new direct numerical simulation model coined E_*n*_PE_*n*_: it allows to solve a set of first principle equations to predict for multiple ions their concentration and electrical potential profiles in electro-chemically complex architectures of *n* layered electrolytes E and *n* polyelectrolytes PE. E_*n*_PE_*n*_ can robustly capture ion transport in sub-millimeter architectures with submicron polyelectrolyte layers. We proof the strength of E_*n*_PE_*n*_ for three yet unsolved architectures: (a) selective Na over Ca transport in surface modified ion selective membranes, (b) ion transport and water splitting in bipolar membranes and (c) transport of weak electrolytes.

Ion transport at interfaces between electrolyte solutions and solid surfaces governs many relevant applications such as electrodialytic and pressure-driven desalination^1^,^2^,^3^, electro-catalysis, batteries^4^, sensors^5^ and micro and nanofluidic applications^6^. The fundamental physico-chemical framework for ion transport in solutions in contact with solid ion sinks such as membranes and electrodes has been initiated by Levich^7^.

However, a wide range of important interfacial transport phenomena is yet to be explained. For example, transport of organic acids in the presence of pH gradients (e.g. in biological systems) is not yet completely understood^8^,^9^. Another example, which is the focus of our work, is the transport in complex layered structures, such as multilayered electrolytes and polyelectrolytes for a wide range of applications.

Experimental ion transport studies have been reported extensively over more than 30 years - mostly for strong electrolytes. The studies can be categorized in (a) fundamental mass transport studies or (b) application studies, the latter being excluded from this background information. Fundamental mass transport studies are mostly performed by measuring classical current voltage relationships. The shape of such IV-curves is frequently affected by concentration polarization phenomenon (CP) which relates to the development of concentration gradients in electrolyte solutions next to ion-selective membranes due to slow diffusion in the boundary layer as compared to more rapid transport in the membrane. Typical concentration profiles are shown in Fig. 1a. Their micrometer scale shape can be resolved experimentally using precise positioning of microelectrodes at various distances from the membrane. Ion distribution at the interface as shown in Fig. 1b however can be only accessed through simulations. Such phenomena happen equally likely at electrodes^7^. The mathematical treatment of the macroscopic diffusion phenomena governing concentration polarization is hence very similar in both systems. However, a detailed description of the interfacial phenomena is much more complex.

While it is widely established that ion transport in such systems is governed by the coupled Nernst-Planck and Poisson equations, the literature mostly involves investigation under too simplified settings. For example, electrolytes are often assumed to be strong, with symmetric ion properties, and unity valence numbers, and geometries are often limited to a single-layer polyelectrolyte sandwiched by an electrolyte^10^.

Leibovitz *et al*. give a historical account on the complexity of real systems and provides a useful list of theorems elucidating the difficulty to expand the understanding towards weak electrolytes^11^. Such questions are of particular importance in, for instance, bioelectrochemical systems, which are subject to multiple physical phenomena like ion transport as well as chemical and electrochemical (dissociation) reactions^12^. Weak electrolytes and their reactions also play a role in new energy conversion systems^13^, where CO_2_ emissions can be used to set up an electric current using ion exchange membranes and porous electrodes. The complex interplay of ion migration and water dissociation has been recently quantified for a porous system by Kamran *et al*.^14^ and gives a taste of intricacy for strong electrolytes. Weak electrolytes behave even more complex. For transport through ion exchange membranes, dissociation reactions of weak electrolytes are anticipated to cause overlimiting currents^15^. The authors suggest that salt depletion leads to a large electric field resulting in a local pH shift within the membrane with the effect that the membrane discharges and loses its ion selectivity.

Other new applications have revealed a lack of understanding of selectivity of multi-layered ion exchange membranes in arbitrary solutions. These multilayer ion exchange membranes have become sophisticated by building Layer-by-Layer architectures as visualized in Fig. 1d. For ion selective membranes, the selectivity can be influenced towards monovalent ions^16^. This is explained by the stronger exclusion of multivalent ions from charged phases according to the Donnan equilibrium^17^. While Abdu *et al*.^16^ report an alternating reversing pattern in mono/bivalent ion selectivity for the sequence of polyanions and polycations, a recent paper of White *et al*.^18^ reports opposite results. PSS/PAH LBL on Nafion cation exchange membranes always show significantly higher selectivities for monovalent ions over bivalent ions. Interpretation of such contradictory results requires a rigorous description of mass transport through multilayer architectures. The parameters of interest in such recent research are the number and thickness of bilayers optimizing mass transfer and permselectivity. Comprehending the influence of a layers thickness, compartment concentration, and applied electric field is yet to be achieved.

Yet other phenomena demonstrate the complexity of such multilayered electrolyte polyelectrolyte systems: Abu-Rjal *et al*.^19^ analytically investigated the influence of boundary layer thicknesses, membrane charge and heterogeneity on the transport numbers of a symmetric 1-1 salt. They find that the convexity of the concentration profile inside a membrane between two adjacent diffusion boundary layers dictates the permselectivity of the system. Garcia-Morales *et al*.^20^ compared iV-curves of a simplified model of multiple layers with experiments and found good agreement, however, did not address questions of selectivity or influence of charge and thickness. Their model is limited and extending such questions to the treatment of multivalent salt mixtures is highly desired but challenging and yet unresolved.

Progress in this field of layered polyelectrolyte structures may also contribute to the understanding of the function of a bipolar membrane, whose application is of importance in separation technology and bioprocessing^21^. Such a bipolar polyelectroyte architecture comprises an anionic polyelectrolyte (aPE) and a cationic polyelectrolyte (cPE) laminated together with a hypothetical junction between the two. Industrially these membranes are used in various large scale applications^22^, however they also enter the field of microfluidic application^23^. pH regulation in micro-chambers can be realized by injecting controllable amounts of protons and hydroxide ions out of the bipolar interface into the microfluidic channel.

With respect to their functionality, the interface of bipolar membranes between the cationic and the anionic polyelectrolyte is of utmost importance. Balster *et al*.^24^ and Abdu *et al*.^25^ experimentally investigated modification of the bipolar junction. Membrane and interface thickness and its influence on the flux and selectivity of bipolar membranes were studied. Until now, such studies remained only experimental and could not be interpreted yet using a single rigorous model over the entire current voltage space.

In this paper, we report a methodology to solve the intricate questions of multi-ion transport through such electrolyte-polyelectrolyte assemblies. We seek to further understand ion selectivity of membranes and evaluate the impact of material and architecture properties on process parameters such as transport numbers and the limiting current density. Direct numerical simulations (DNS) allow access to concentration and potential distributions in a layered system with enforced background charge under the influence of an electric field. By varying the electric field, the composition of the solution and the geometry of the system, the influence of these parameters is estimated. For the large variety of different architectures, this unified approach establishes a new framework to address and interpret yet inaccessible architecture properties. The framework is accessible through a web page^26^.

## Results

In the following, we will demonstrate that our framework can resolve questions such as

- The prediction of ion selectivity of layer-by-layer modified electrodialysis membranes for mono- and bivalent ions.
- The prediction of the rate of water splitting in a bipolar membrane and the associated iV-curve.
- The prediction of the current density as a function of driving force for a weak electrolyte such as a mono- or bifunctional organic salt.

### Selectivity of a surface modified membrane

The simplest example of a surface modified membrane is a cation exchange membrane (CEM) with an added anion exchange polyelectrolyte layer (aPE). The CEM is for instance a commercially available membrane with known thickness and charge density. The latter properties are usually unknown for any added polyelectrolye layers due to the uncertainty of the deposition process. Consequently, little is known about the influence of charge density and thickness on mass transfer and selectivity. It is assumed that the thickness of a deposited layer is in the order of 1–100 nm, however experimental determination is often difficult on real ion exchange membranes. Data on the charge density are also lacking, especially since the deposition process could alter the integrity of the original surface or any previously deposited underlying polyelectrolyte layer in the case of multiple layer-by-layer deposits.

The presented direct numerical simulation tools can be used to examine different parameters (e.g. layer thickness or charge) over a wide range of uncertainty gaps. Comparison of these simulations with standardized experiments can then be used to narrow the uncertainties of the fabrication process. Below we present an example of such calculations in which sensitivity of Sodium selectivity is predicted as a function of design parameters.

The following E_*n*_PE_*n*_ simulation considers an aqueous 1:2 mixture of Calcium- and Sodium chloride, such that the overall concentration of positive charge is 1 mM in the reservoir connecting to the CEM layer. Two diffusion boundary layers of 100 *μ*m are formed on each side of a surface modified membrane. The CEM was set to be 100 *μ*m thick comprising a charge density of 1 M. The charge density and thickness of the aPE and the potential drop across the system were altered, yielding their influence on the sodium selectivity. It has to be noted, that these parameters interdependently alter the charge behaviour and therefore the results can not be attributed to one parameter alone. The sign of the electric field is set across the membrane such that the permeating species has to first pass the layer of same charge. Due to the Donnan equilibrium and the correlated charge exclusion, multivalent ions are rejected to a higher degree than monovalent ions.

**Figure 2:**
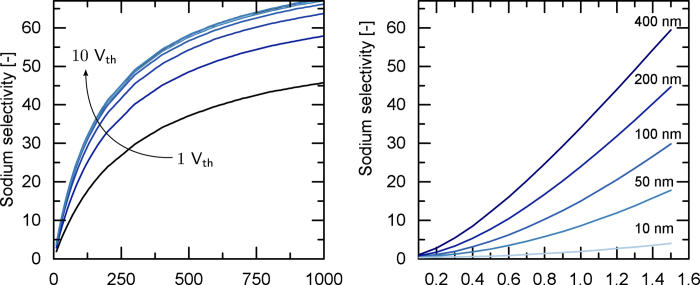
Selectivity of Sodium over Calcium as a function of **a**) polyelectrolyte layer thickness and potential drop for a charge density of 0.5 M and **b**) aPE charge density for 1 V_*th*_.

Figure 2a shows the Sodium selectivity,


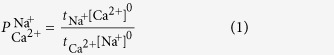


of the modified membrane for varying aPE thickness and potential drop. Here, the fraction of the transport numbers of sodium and calcium is normalized by the fraction of their reservoir concentrations. The aPE charge density was chosen to be 0.5 M. As the aPE layer thickness increases, the relative magnitude of calcium rejection over sodium also increases, which leads to improved sodium selectivity. However, the gain in selectivity slows down when the aPE thickness gets above a few hundred nm. Differing layer sizes also result in different resistances, which may further complicate comparison. The plateau of the curve with thickness indicates a trade-off between improved selectivity and resistance, showing potential in optimizing LbL fabrication.

Figure 2b captures the influence of aPE charge density for varying aPE thicknesses. The potential drop across the system was chosen to be 1 V_th_ ≈ 0.025 *V*. Increasing the charge density will lead to a stronger rejection of multivalent ions compared to monovalent ions and thus to a higher Sodium selectivity. It can be concluded that the aPE charge density and thickness are crucial for the permselectivity, however, the thickness loses it’s strong influence above a certain limit. For the system considered in Fig. 2a, the optimal trade-off between resistance and selectivity is achieved for a aPE of 70 nm. When applying 10 V_th_ the transport number for Sodium will be 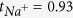.

**Figure 3:**
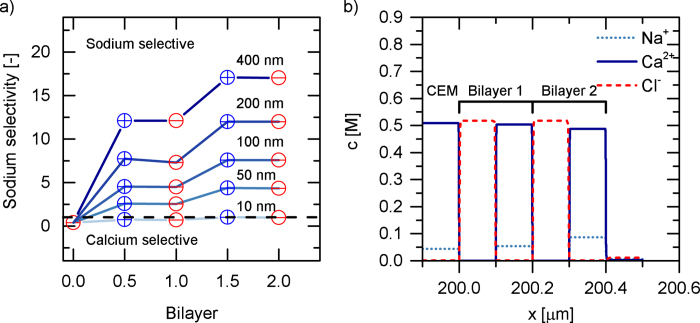
**a**) Selectivity of Sodium over Calcium as a function of numbers of layers **b**) Concentration profiles of ions in a two-bilayer assembly.

Figure 3a shows simulation results of a CEM modified with two bilayers, where one bilayer comprises one cPE and one aPE. Sodium selectivity generally increases with increasing number of layers. Experimental data of White *et al*.^27^ is in agreement with these simulations, the experimental data reported by Abdu *et al*.^25^ only partly agree with the model. Discrepancies arise in the observed decrease of selectivity for added cPE layers. While the simulation predicts no or only slight selectivity difference, the experimental data suggests a considerable decrease. With the E_*n*_PE_*n*_ simulations at hand and the new supporting data by White *et al*.^27^, we now hypothesize that the deposition of an additional aPE layer reduces the integrity of the underlying cationic layer. Defects in this layer will spoil the mechanism of Donnan exclusion, effectively decreasing overall selectivity towards monovalent cations. Depositing more alternating layers of cation and anion exchange slowly pushes selectivity to an overall monovalent ion selectivity. If one would succeed to prepare stable fixed aPE layers, equally good selectivities or even better experimental results can be achieved using one layer with defined thickness and high charge density.

Figure 3b exemplarily shows the concentration profiles in the two bilayers of a modified CEM with no applied potential. The concentration ratio of Calicum/Sodium in cationic layers is 5, while the mobility ratio is 0.6. In the aPE layers, the concentration ratio is 0.0142, which governs the selectivity of the system. When applying an electric potential, the state of the boundary layers will change and the system will transition to be Sodium selective.

### Water splitting inside a bipolar membrane

A bipolar membrane is at least a triple layer architecture of an anion exchange (aPE) and a cation exchange membrane (cPE) enclosing a significantly thinner junction layer. The nature of the junction layer is often tailored to improve its functionality. Upon applying an electric field, complex mass transport and water splitting behaviour emerges. The exact thickness of the junction depends on the manufacturing process and can’t be measured directly, but is believed to be in the order of nanometers^28^. It is understood that the junction dictates the water splitting kinetics, proven by various efforts to manipulate its properties, e.g. by doping with catalytic materials.

The influence of bipolar membrane properties on mass transport behavior has been subject to significant experimental and theoretical efforts. Volgin and Davydov^29^ have modelled a bipolar membrane and examined the development of concentration profiles as a function of time. The influence of membrane charge, bulk concentration and geometry on complete iV-curves has not yet been investigated and hence comprehensive interpretation of experimental observations remains impossible. A typical experimental observation is the presence of two limiting current densities. At low current densities, when starting from the salt form of the bipolar membrane, a first limiting current density can be observed which is directly related to the co-ion leakage through the bipolar membrane. Ample experimental evidence has been established showing that this *i*_lim_ can be tuned through the thickness and the charge density of the monopolar parts of the bipolar membrane^30^. A second limiting current density occurs at much higher current densities and is interpreted to be related to an imbalance in high water splitting rates and significantly lower diffusional water supply into the bipolar junction^31^.

The E_*n*_PE_*n*_ framework can be set up to simulate a bipolar membrane; two oppositely charged polyelectrolyte layers separated by an electrolyte junction layer are enclosed by two diffusion boundary layers. The polyelectrolye layers will be given the properties of typical technical CEM and AEM membranes, i.e. a thickness of 100 *μ*m and a charge density of 1 M. Junction thicknesses in the range of 1–10 nm are considered. The system comprises NaCl in water, where water can split into H^+^- and OH^−^-ions, consuming H_2_O, according to the autoprotolysis reaction. The forward reaction rate is chosen sufficiently high such that equilibrium is attained. The backward reaction rate can be derived from the water product *K*_*w*_ = 10^−14^. The oppositely charged membranes will lead to a high electric field in the junction layer, promoting ion transfer. Due to the charge exclusion of Sodium and Chloride, the only source of ions in this region is the water splitting reaction. In applications this can be used to produce acids and bases from their respective salt. In our current approach, additional complexity in the kinetics of the autoprotolysis reaction are neglected. Such complex phenomena may be a field enhanced water splitting mechanisms and catalytic interactions with the membrane material^32^,^33^,^34^,^35^. Our simplification of water splitting kinetics can be justified based on recent findings of Andersen *et al*.^36^ and their confirmation that under steady-state regimes the chemical non-equilibrium effects do not play a significant role.

**Figure 4:**
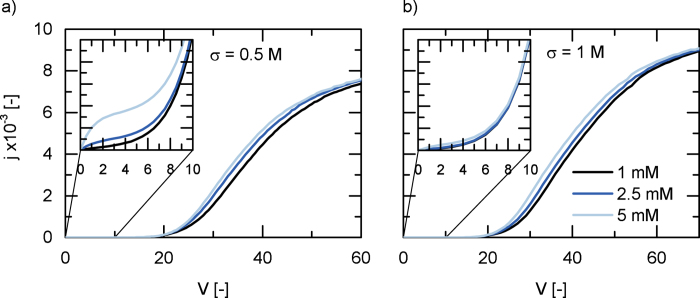
Current-voltage behaviour of a BPM comprising a 2 nm junction for varying concentrations of salt. Membrane charge density is *σ*_AEM_ = *σ*_CEM_ = a) 0.1 M b) 1 M.

Figure 4 shows current voltage curves for varying salt concentration in the bulk liquid and various membrane charge densities. The onset of water splitting and related increase in current density is visible at 20 V_th_. For a chosen potential, the overall potential drop can be attributed to the drop across the two boundary layers and the junction. The fraction of the overall drop occuring in each region is determined by the conductivity of the corresponding electrolyte solution. The conductivity in the junction layer can be assumed constant, since there will be almost no salt ions present and the autoprotolysis of water is close to equilibrium. The boundary layer conductivity increases with the bulk concentration of salt. Consequently, the potential drop across the junction layer increases as well. This results in an slightly earlier onset of water splitting, indicated by the offset of the current voltage curves in both diagrams.

The inserts in Fig. 4 show the lower part of the current voltage curve. The current evolving at low electric potentials is due to the co-ion leakage of the individual membranes. The leakage directly relates to the Donnan exclusion, which is prominent in highly charged membranes. It can be seen, that for a bipolar membrane of 1 M charge density, co-ion leakage is almost completely suppressed while a less selective membrane shows more co-ion leakage increasing also with increasing ion concentration in the bulk solution. The modelled phenomena compare well to experimental results^37^.

**Figure 5:**
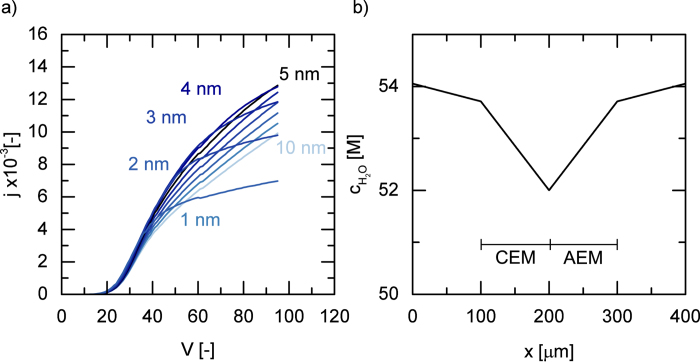
**a**) Current-voltage behaviour of bipolar membranes depending on the junction thickness. The onset of water splitting at V = 20 V_*th*_ is common for all junction thicknesses, the current behaviour at higher potentials exhibits strong non-linear dependence on junction thickness. **b**) Concentration profile of Water over the domain at limiting current density. At the 2 nm junction water concentration is only slightly reduced.

With respect to the analysis of the second limiting current density, the model can be used to investigate the effect of junction thickness for instance. Figure 5 indicates that features of the current voltage curves depend strongly on the junction thickness for high potential drops. The onset of the limiting current density moves with increasing junction thickness towards higher current densities and higher potentials. The total rate of H^+^ and OH^−^ ion production in the junction layer is the integral of the reaction rate over the junction volume. Therefore, a larger volume explains a higher limiting current density. However, sustaining a large electric field over thicker junction layers also requires a larger overall potential drop. In the limit of vanishing a junction thickness, the potential drop will overlap with the membranes, however, behaving similarly to a 1 nm junction. Consequently, for a given production rate there exists an optimal junction layer thickness. In the example of Fig. 5 for current densities below 0.012, a junction thickness of 4–5 nm would minimize power consumption. The results show that the proposed autoprotolysis kinetics do not lead to a complete drying of the membrane junction. Here, the limiting current density stems from a slight decrease in available water ions. Concentration profiles, pH and the development of the corresponding iV curve are visualized in a supplementary video.

### Weak electrolytes

Weak electrolytes play a major role in bioprocessing and bioengineering. Ionic transport coupled with dissociation governs many applications, e.g. artificial organs, down-stream processing of biotechnological conversions and drug delivery. While pH dynamics in membrane boundary layers have recently attracted significant attention^36^,^38^, its application to a more general geometry has not yet been reported.

In the following, a single AEM is used to selectively transport the anion of a weak 1-1 salt in the presence of water ions. The full set of reaction is


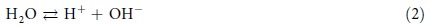



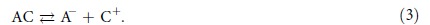


Such a system is a first step to represent more complex mixtures. The salt dissociates according to an equilibrium constant into an anion and a cation. At underlimiting currents and neglecting water dissociation, our simulations predict ion concentration profiles in the electrolyte precisely as an analytical model presented in the Methods section. With increasing currents however, the contributions of H^+^ and OH^−^ ions to the overall transport rate can become significant and local pH changes may occur. Such local pH changes can only be tracked by our simulation since H^+^ and OH^−^ ion concentrations are included in the modelling.

**Figure 6:**
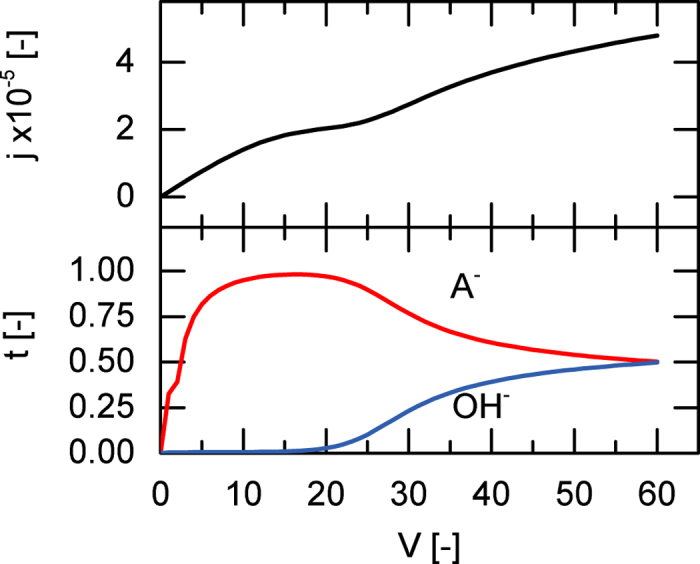
Current-voltage behaviour of a weak 1-1 salt with pK = 4. At *V* = 40 *V*_*th*_, decreasing pH in the feed shifts the dissociation equilibrium towards the anion, which decreases overall resistance. The transport numbers are sampled at the membrane interface.

Figure 6 shows the current voltage curve featuring multiple resistances. The first limiting current density can be attributed to the depletion of the salt on the feed side of the membrane. Once the voltage reaches approximately 20 *V*_th_ or 0.5 V, the additional flux of H^+^ and OH^−^ ions leads to a lowered resistance. This is confirmed by the increase in transport number of OH^−^ sampled at the membrane interface. At higher potentials, both counter-ion transport numbers approach roughly 0.5.

**Figure 7:**
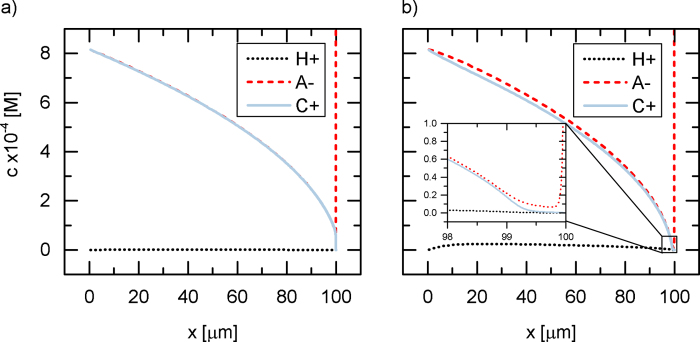
Concentration profiles in the depletion boundary layer for a weak salt at potentials **a**) 37 V_*th*_ and **b**) 60 V_*th*_.

Figure 7 shows the concentration profiles of a weak salt in steady state at 37 V_th_ and 60 V_th_. At the lower potential, pH = 7 and the cation and anion concentration match. After the onset of water splitting, the concentration of H^+^ has significantly increased and offsets the salt’s dissociation equilibrium towards the anion, by means of the electric field maintaining electroneutrality. A similar effect is described for weak acids as the barrier effect^39^. At *x* = 100 *μ*m the anion concentration increases rapidly due to the positively charged membrane, which continues to 200 *μ*m and has a fixed charge concentration of 1 M. It can be seen that at 37 V_*th*_ the salt is almost completely depleted and at 60 V_*th*_ an extended space charge region has formed. A supplementary video shows the concentration profiles of ions for the full range of voltages.

The trend of increasing H^+^ concentration in the depletion compartment as seen in Fig. 7b was also predicted by a simplified model^40^. Even though they investigated carbonic acid, we can still compare the results to a weak salt system as both cases are occurring at pH = 7. However, partition coefficients were assumed to predict anion concentrations in the membrane phase^40^.

Our simulated current voltage curves also compare favourably to experimental results^41^ for Nafion 117 and solutions of iron- and sodium sulfate. The occurrence of an intermediate limiting current density as shown in Fig. 6, was also observed^41^. This intermediate limiting current density occurs at approximately 0.5 Volts (or 20 V_*th*_). The overlimiting current is attributed to electroconvection^41^.

## Discussion

We have shown that the E_*n*_PE_*n*_ framework can predict all important one-dimensional phenomena in electrically driven membrane processes. The approach is sufficiently general to simulate layer-by-layer assemblies, bipolar membranes and reacting electrolyte solutions of weak electrolytes. For the first time, a coherent reference model has been established that can help experimentalists decide whether to attribute an experimental observation to known phenomena described by our approach or to take into account deviations of their system (e.g. membrane heterogeneity).

The framework can easily be extended. Initial work shows that the introduction of convective flow into the species flux equation allows the unprecedented possibility to couple convection, diffusion and charge controlled transport of multiple charged and uncharged species in layered electrolyte and polyelectrolyte systems. This could potentially resolve many of the open question on renal salt, protein and solvent transport through the glomerular barrier - the latter comprising layers of podocytes and endothelial cells sandwiching a highly charged biomacromolecular layer^42^. A more advanced extension could include two-dimensional effects and geometries. Specifically, combining the Navier-Stokes equation, to capture possible electroconvective motion^43^,^44^,^45^, with EnPEn will provide a unified platform to assess importance of various effects in ion transport in membrane systems.

Extending today’s steady state version of E_*n*_PE_*n*_ into a dynamical model would allow the observation of transient phenomena. In fact, transient hysteresis in ion transport phenomena is discussed in recent literature^46^,^47^; the extent of transport hysteresis is scan rate and ion mobility dependent. This suggests that the appropriate time dependent electrical potential forcing could induce dynamic ion selectivity, for complex electrolytes with ions of the same charge but different mobilities due to differences in valence numbers or size. This is likely to be of practical relevance in microfluidic and lab-on-a-chip devices. Development of a dynamical version of E_*n*_PE_*n*_ is challenging, however not impossible considering the versatility of the numerical tools used so far.

E_*n*_PE_*n*_ is flexible and versatile: by changing boundary conditions, adding mass transport contributions, reaction terms and introducing models to predict ionic activity coefficients, the prediction of complex systems such as ion selective electrodes^48^, bioelectrochemical systems^49^ and nanofluidic systems^50^ come into reach. One particular feature of membraneous layers in cellular barriers is their pH dependent charging behaviour due to their amino acids having acidic and base functional groups. This can be modelled in E_*n*_PE_*n*_ by allowing a pH dependent dissociation reaction for the fixed charges on the polyelectrolyte. Furthermore a convection contribution needs to be added to ion transport equation.

## Methods

The model section comprises two major parts: (a) the explicit description of all equations governing the phenomena occuring in the layered archtitectures of elelectrolytes and polyelectrolytes and (b) the validation of the model and specific modifications to incorporate dissociation reactions.

### Model description

We follow the description of a trilayer system with a charged membrane adjacent to two stagnant diffusion layers as described by Andersen *et al*.^36^. For steady state conditions the dimensionless set of equations for *N* species include


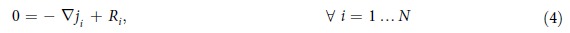



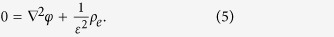


The first equation represents conservation of ionic species, in which *j*_*i*_ is the flux of species *i* described by the Nernst-Planck equation,


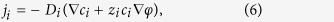


where *c*_*i*_ is the concentration of the *i*^th^ with *z*_*i*_ being its charge and *D*_*i*_ its diffusion coefficient, and *φ* the electric potential in thermal units 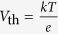. The first term on the right hand side of eqn. (6) represents the diffusive mass flux and the second term represents the ion electromigration flux. Here it is assumed that activity coefficients are unity and the difference in the reference chemical potential of membrane and solution phase are negligible. These assumptions hold for membranes with high water content and diluted solutions. *R*_*i*_ are the source terms, derived from the reactions specified for a given system. In the Poisson equation *ρ*_*e*_ is the free electric charge density,


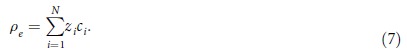


*ε* is a dimensionenless length scale, resembling the Debye-length,


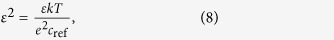


where, *ε* is medium electric permitivity, *k* is the Boltzmann’s constant, *T* is the absolute temperature, *e* is the charge of a proton, and *c*_ref_ is a reference concentration (number per unit volume) in which all other concentrations are expressed. In the membrane, the diffusion coefficient is altered by a factor *α* < 1, to account for effects such as porosity and tortuosity (here we used *α* = 0.2), and a fixed background charge is added to the charge density,


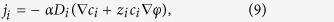



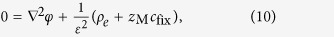


where *z*_M_*c*_fix_ is the fixed charge concentration of the ionic groups, assuming a strongly dissociating membrane group.

The set of equations is non-dimensionalized by choosing *c*_ref_ = 1 M. Additionally, to avoid very small or very large numbers, we express lengths in microns and time in milliseconds.

The system of equations is solved on non-uniform, staggered meshes. Second order central difference schemes are used to compute fluxes at the faces of control volumes. The resulting discretized system is fully conservative, and does not use artificial smoothing or numerical filter to treat sharp gradients. The coupled discretized equations are solvied in one dimension perpendicular to the membrane using a Newton line search implementation of the iterative, non-linear solvers by PETSc^51^ on a high resolution mesh. The boundary conditions are provided by the composition and potential in the feed and receiving compartment.

The implementation was validated using data from literature (c.f. Supplemental Material) and comparisons with analytical derivations of concentration profiles for weak electrolytes and limiting current densities for multivalent ionic mixtures.

### Model extension to dissociation reactions

In this subsection, an analytical model predicting the concentration profile of a weak salt in an electroneutral boundary layer of a charged membrane at limiting current density is derived. The model is then used to numerically validate the addition of the source term describing reactions in the mass convervation equation.

The weak salt dissociates according to an equilibrium constant to equal amounts of one-valent anions and cations,


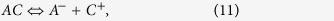



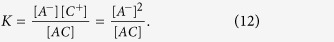


The transport of species in this layer of thickness *L* can then be described by a Nernst-Planck equation in dimensionless formulation for each of the species


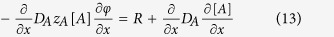



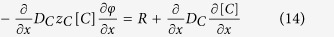



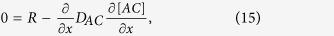


where *R* is the chemical source term for the dissociation reaction. Assuming a scenario in which electroneutrality holds and assuming a symmetric electrolyte with *D*_*A*_ = *D*_*C*_, one can add equations for all elements and write


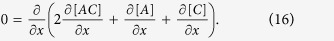


By inserting eqn. (12) in eqn. (16) and integrating, the concentration profile of [*A*] in the boundary layer depending on integration constants *c*_1_ and *c*_2_ is obtained:


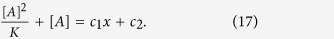


By choosing [*A*](*x* = 0) = *c*_*res*_ and [*A*](*x* = *L*) = 0,


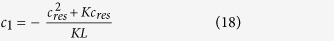



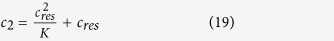


With 
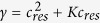
, the final profile and its fist derivative are


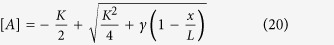



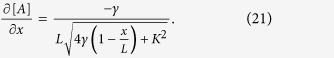


**Figure 8:**
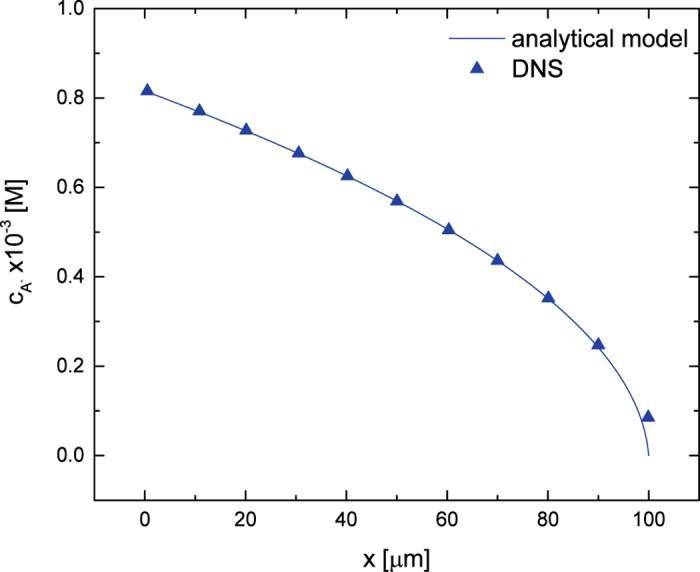
Comparison of ion concentration profiles predicted by the analytical model and DNS for a weak 1-1 salt with pK = 4.3.

As shown in Fig. 8 the DNS code prediction is in excellent agreement with this analytical solution. The deviation of the DNS results from analytical model is visually indeterminable with the exception of the region close to the membrane, where the DNS deviates due to the explicit modelling of the membrane, whereas the analytical model assumes complete ion depletion.

## Additional Information

**How to cite this article**: Femmer, R. *et al*. Ion transport through electrolyte/polyelectrolyte multi-layers. *Sci. Rep*. **5**, 11583; doi: 10.1038/srep11583 (2015).

## Supplementary Material

Supplementary Information

Supplementary Video S1

Supplementary Video S2

## Figures and Tables

**Figure 1:**
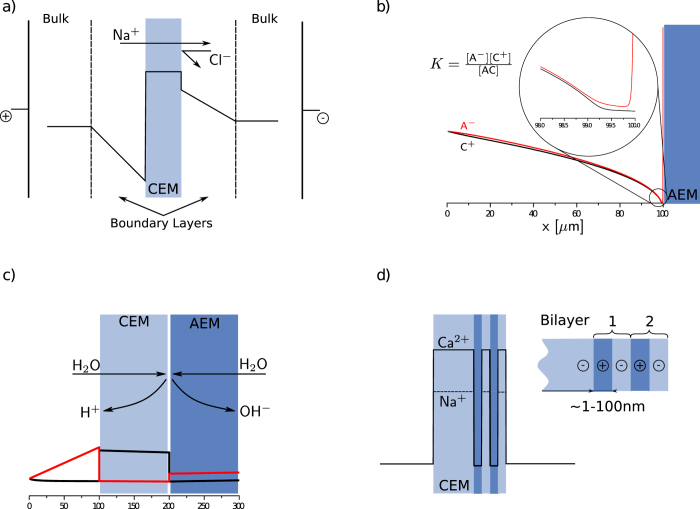
Graphical representation of the architectures described with the new (E_*n*_PE_*n*_) model of n electrolytes E_*n*_ and n polyelectrolytes PE_*n*_ and a number of occuring phenomena. Shown are the (**a**) principles of electrodialysis for desalination, (**b**) concentration profiles for weak electrolytes in the concentration polarization layer, (**c**) the principle of a bipolar membranes for acid and base production and (**d**) selectivity in layer-by-layer modified membranes.
