# Supplementary material for: Ion transport through electrolyte/polyelectrolyte multi-layers
Source: Sci Rep. 2015 Jun 26;5:11583. doi: 10.1038/srep11583 (PMC4481379; doi:10.1038/srep11583)
Supplement: Supplementary Information [file srep11583-s1.pdf]

Ion transport through electrolyte/polyelectrolyte multi-layers

Robert Femmer*, A. Mani† and M. Wessling*‡

May 14, 2015

Model validation To validate the model, a symmetric trilayer with a cation exchange membrane in the middle was simulated in steady state with increasing potential drop and no reactions. The resulting current voltage iV-curve was compared to simulations in literature[1]. However, the systems are not entirely equivalent since the reference simulation used an idealized membrane model as a perfectly selective boundary condition with fixed counter-ion concentration equal to twice that of the reservoir. In contrast, our simulations fully resolve the membranes without any assumption on interfacial counter-ion concentration or selectivity. For a meaningful comparison, the potential drop was sampled between the bulk side and wherever the counter-ion concentration reaches approximately 2 in the depletion layer close to the membrane. To simulate the behaviour of an ideally selective membrane, the fixed charge concentration c_m was set to a multiple of the bulk concentration. As shown in Fig. 1, the deviation can be decreased by further increasing the membrane charge and downsizing of the membrane and enrichment layer thickness.

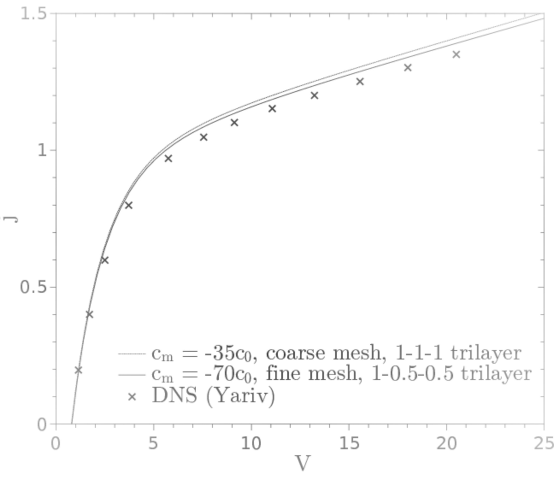

Figure 1: Comparison with data in literature[1].

*AVT Chemical Process Engineering, RWTH Aachen University, Turmstr. 46, 52064 Aachen, Germany
†Department of Mechanical Engineering, Stanford University, Stanford, CA 94305, USA
‡DWI, Forckenbeckstr. 50, 52074 Aachen, Germany

Code structure To reproduce or modify the numerical simulations shown in this paper, a few files need to be edited:

- meshdata/*-1D.geo
- sysdata/*.sys
- bpm.c, said.c, main.c
- Makefile

The *-1D.geo files are files parseable by the open source software Gmsh and make use of the boundary layer fields around the membrane interfaces. Gmsh translates these files into *.msh mesh definitions, which determine the spatial discretization used by the Finite Volume method. An example for a 100 μm membrane with an attached PE Layer of 1 μm follows. The enclosing boundary layers are 100 μm each. The points lie on one line, constructing one-dimensional zones, having the width of the line between the points. Boundary layers are constructed around interfacial points, allowing the resolution of space charge regions.

```
lc = 1;      // 1 mu
dbl = 100;  // boundary layer thickness [mu]
memb = 100; // membrane thickness [mu]
poly = 1;   // PE layer thickness [mu]
Point(1) = {0.0, 0.0, 0.0, 0.1};
Point(2) = {dbl, 0.0, 0.0, 0.1};
Point(3) = {dbl+memb, 0.0, 0.0, 0.1};
Point(4) = {dbl+memb+poly, 0.0, 0.0, 0.1};
Point(5) = {dbl+memb+dbl, 0.0, 0.0, 0.1};
Line(1) = {1,2};
Line(2) = {2,3};
Line(3) = {3,4};
Line(4) = {4,5};

Field[1] = BoundaryLayer;
Field[1].NodesList = {2, 3, 4};
Field[1].hfar = 0.1;
Field[1].hwall_n = 0.0002;
Field[1].ratio = 1.01;

Background Field = 1;
```

The *.sys files define the aqueous solution and the reactions therein. The files are supplied to a python script implementing a lexical parser and compiling it to a *.def file resembling the C-struct of the simulation software. The keywords are "S", "G" and "REAC". "S" is followed by an identifier of the species, it's molar weight, diffusion coefficient in water and hydration number. The molar weight and hydration number are not yet in use. "G" is followed by the species' identifier and a list of UNIFAC groups, which are defined in sys/defgen.py. The specific groups are not in use, however the charge of the species is derived from the sum of the charges of the groups. The number of participating groups can be altered by prepending the number to the group name, e.g. "G H2O 1H2O". The "REAC" keyword is to be followed by a forward reaction constant, e.g. "f=1e2;" and an equilibrium constant "k=14;" or backward reaction constant "b=1e-12;". Then a list of species prepended with the stoichometric coefficient follows, e.g.

"REAC f=1e2; k=14; -1H+ -1OH-" would define a water splitting reaction, assuming the H2O concentration remains constant. An example for glutamic acid follows.

```
# Filename: glutamic_acid.sys
# Id    MW      Diff.-coeff Hydration number(unused)
S H2O   18.5    1e-10       0
S H+    1       9.3e-09     0
S OH-   17      5.3e-09     0
S GLU-- 145.13  6.55765e-10 0
S GLU-  146.13  6.53518e-10 0
S GLU0  147.13  6.51293e-10 0
S GLU+  148.13  6.49091e-10 0

# Group definitions
G OH-       1OH-
G H+        1H+
G H2O       1H2O
G GLU--     2COO- 1CH2 1NH2
G GLU-      1COO- 1COOH 1CH2 1NH2
G GLU0      2COOH 1CH2 1NH2
G GLU+      2COOH 1CH2 1NH3+

# Reactions
REAC f=1e2; k=14;   -1H+ -1OH-
REAC f=1e2; k=4.32; 1GLU- -1GLU-- -1H+
REAC f=1e2; k=2.16; 1GLU0 -1GLU- -1H+
REAC f=1e2; k=9.96; 1GLU+ -1GLU0 -1H+
```

The C files contain the main function of the program. The numerical simulation is set up by defining membranes and their charge. Examples on how to define the struct values can be found in modules.h. The *.def files are loaded into the param_t struct by calling the param_set function.

The file simulations.h defines some convenient interfaces to the solver, e.g. the function sweep_boundary_chargeall() will, for the supplied parameter, range and stepsize, drive up the charges of all zones to their respective end_charge. This can be used to simulate charged membrane systems without knowing suitable initial values.
The output will be saved to a file specified by the app_set_name() function and reside in the outdata-directory. The files can be inspected by the python2 scripts plot.py, which draws profiles over the domain and ivc.py, which draws profiles over the sweep range, e.g. potential. The code is available upon request.

Videos The supplementary material includes two videos showing steady state concentration profiles as a function of potential; the first shows a bipolar membrane surrounded by a 1mM NaCl-solution. The left-most graphs show the concentration profiles of NaCl-ions, while the potential drop over the domain is increased. The bottom figure is a zoomed view of the above. At lower potentials, salt ions are slowly replaced by water ions, whose concentrations are rising due to increased water splitting taking place in the membrane junction. This is also shown by the pH profile in the bottom right graph. At higher potential drops, uncharged water is starting

to deplete from the membrane junction.

The second video shows an anion exchange membrane surrounded by a weak salt solution with pKa=4.3. The limiting current density is clearly related to the depletion of salt ions from the feed compartment (left). An increase of water splitting can be seen in the offset of A- and C+ concentrations in both boundary layers and the pH profile.

It is possible to try out simulations online [2].

References

[1] Yariv, E. Asymptotic current-voltage relations for currents exceeding the diffusion limit. *Phys. Rev. E* **80**, 051201 (2009).

[2] Femmer, R. & Wessling, M. Enpen simulations online (2015). URL `http://enpen.avt.rwth-aachen.de/`. Date of access: 14/05/2015.

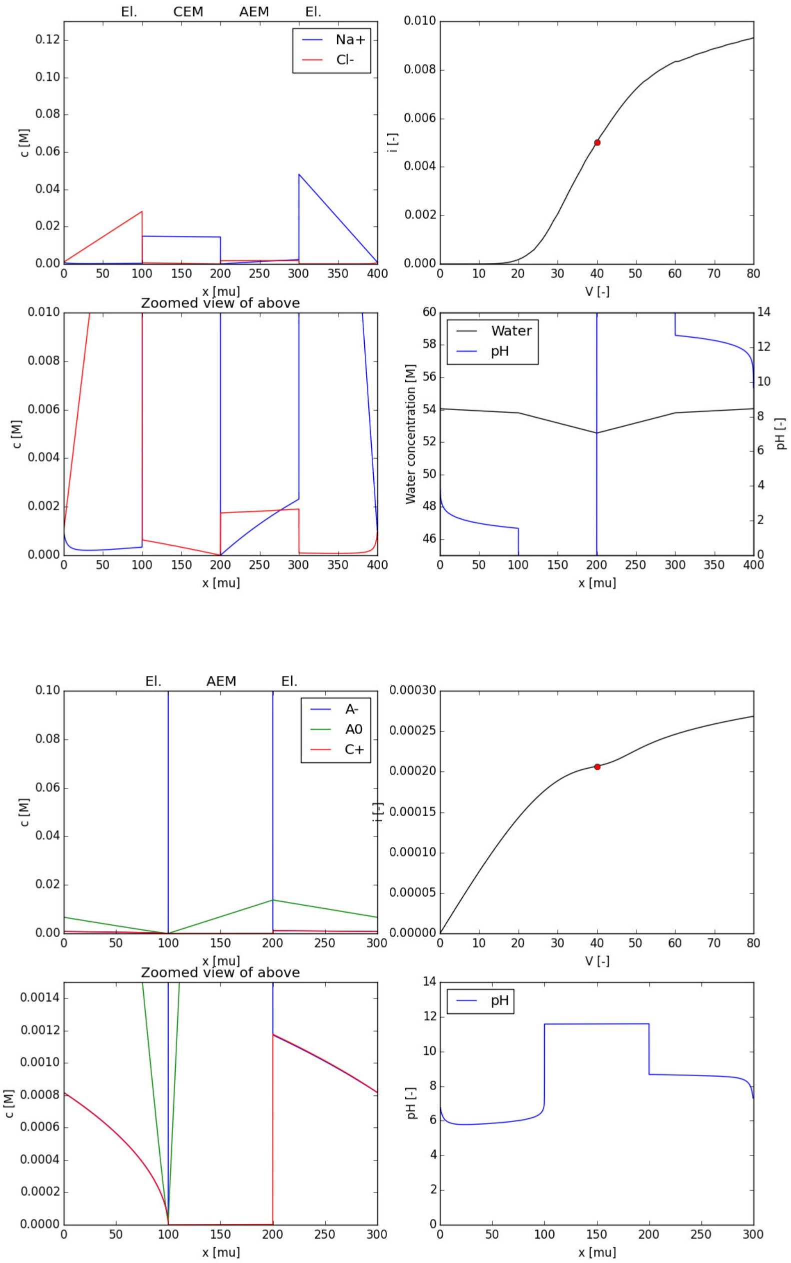

Figure 2: Screenshots of the two videos supplied, showing concentration profiles as a function of potential drop for a bipolar membrane/salt system and anion exchange membrane/weak salt system.
